# Effect of β − hydroxy − γ -aminophosphonate (β − HPC) on the hydrolytic activity of *Nocardia brasiliensis* as determined by FT−IR spectrometry

**DOI:** 10.3389/fmicb.2023.1089156

**Published:** 2023-01-26

**Authors:** Sandra Martínez-Robles, Erik González-Ballesteros, Jorge Reyes-Esparza, Isaí Trejo-Teniente, Blanca Estela Jaramillo-Loranca, Alejandro Téllez-Jurado, Víctor H. Vázquez-Valadez, Enrique Angeles, Genaro Vargas Hernández

**Affiliations:** ^1^Departamento de Ciencias Biológicas, Facultad de Estudios Superiores Cuautitlán, Universidad Nacional Autónoma de México, Cuautitlán Izcalli, Mexico; ^2^Programa Educativo del Posgrado en Biotecnología, Universidad Politécnica de Pachuca, Zempoala, Mexico; ^3^Facultad de Farmacia, Universidad Autónoma del Estado de Morelos, Cuernavaca, Mexico

**Keywords:** FT-IR, *Nocardia brasiliensis* spectrum, L-carnitine analog, hydrolysis, casein, L-tyrosine, Tween 80, egg yolk

## Abstract

The use of immunomodulatory and metabolic modulating drugs has been considered a better strategy to improve the efficacy of conventional treatments against pathogens and metabolic diseases. L-carnitine is relevant in fatty acid metabolism and energy production by β-oxidation, but it also has a beneficial therapeutic immunomodulatory effect. The β-hydroxy-γ-aminophosphonate (β-HPC) was developed, synthesized and studied in different pathologies as a more soluble and stable analog than L-carnitine, which has been studied in bacterial physiology and metabolism; therefore, we set out to investigate the direct effect of β-HPC on the metabolism of *N. brasiliensis*, which causes actinomycetoma in Mexico and is underdiagnosed. To analyze the effect of β-HPC on the metabolic capacity of the bacterium for the hydrolysis of substrate casein, L-tyrosine, egg yolk, and tween 80, Fourier transform infrared spectroscopy (FT-IR) was employed. It was found that β-HPC increases the metabolic activity of *N. brasiliensis* associated with increased growth and increased hydrolysis of the substrates tested. By the effect of β-HPC, it was observed that, in the hydrolysis of L-tyrosine, the aromatic ring and functional groups were degraded. At 1515 cm^–1^, any distinctive signal or peak for this amino acid was missing, almost disappearing at 839, 720, 647, and 550 cm^–1^. In casein, hydrolysis is enhanced in the substrate, which is evident by the presence of NH, OH, amide, and CO. In casein, hydrolysis is enhanced in the substrate, which is evident by the presence of NH, OH, amide, COO, and *P* = O signals, characteristic of amino acids, in addition to the increase of the amide I and II bands. In Tween 80 the H-C = and *C* = C signals disappear and the ether signals are concentrated, it was distinguished by the intense band at 1100 cm^–1^. Egg yolk showed a large accumulation of phosphate groups at 1071 cm^–1^, where phosvitin is located. FT-IR has served to demonstrate that β-HPC is a hydrolysis enhancer. Furthermore, by obtaining the spectrum of *N. brasiliensis*, we intend to use it as a quick comparison tool with other spectra related to actinobacteria. Eventually, FT-IR may serve as a species identification option.

## 1. Introduction

*Nocardia brasiliensis* is a Gram-positive, acid-fast variable, catalase positive, oxidase negative, a strictly aerobic bacterium. It grows thin and branched filamentous that easily mimics the fungal mycelium. The colonies are confluent layers embedded on the agar surface, powdery white to yellow on the obverse and orange on the reverse side. To obtain developed colonies, 72 h to 15 days must elapse. In addition, a “wet soil” aroma scents the environment in full-grown. *N. brasiliensis* commonly inhabits agricultural soils due to its abundance of organic matter; it has a genome of 9436348 bp enriched through a horizontal transfer with genomes from saprophytic bacteria and insects. This has contributed to the bacterium expressing the following virulence factors: hemolysins, catalases, superoxide dismutases, proteases, lipases, and cell wall lipids such as trehalose monomycolate and 6,6, dimycolil trehalose (de Chastellier, 2009; Trevino-Villarreal et al., 2012; Vera-Cabrera et al., 2013). In addition to allowing their establishment in the host, these lipids attract macrophages and dendritic cells that begin to produce and secrete TGF-β and IL-2, thus establishing immune tolerance to accidental intracellular pathogen. Like *Mycobacterium tuberculosis*, *N. brasiliensis* infects macrophages and induces granulomatous responses. In Mexico, it is inoculated through traumatic skin lesions and causes actinomycetoma, a chronic infection of the skin and underlying tissues that usually affects the bones, causing relatively painless enlargement and fistulas through which pus and “grains” formed by bacterial filaments are discharged, *N. brasiliensis* is isolated in the majority cases (65.6%) (López-Martínez et al., 2013; Emery and Denning, 2020). Since poverty is the main characteristic associated with the development of the disease, coupled with possible cellular immunocompromised (Mahgoub, 1978; Bonifaz et al., 2014), dissemination and conversion to systemic nocardiosis are possible, which could endanger the patient’s life. Accurate diagnosis is achieved by isolation in enriched media such as Lowenstein–Jensen, brain heart infusion, and blood agar. Followed by hemolysis, hydrolysis, and antimicrobial sensitivity tests. Contaminants can develop first if there is no pure strain, especially in primary insulation, hence the importance of ensuring bacterial growth or developing better diagnostic strategies, as misdiagnosis results in failed treatment. An advantage in identifying the genus Nocardia is that it grows in the patient and forms compact clusters called grains, which makes it possible to perform PCR without need for isolation. Similarly, 16S rRNA can be sequenced and another alternative is the identification of plasmids by pulsed-field gel electrophoresis (PFGE). Likewise, matrix-assisted laser desorption/ionization mass spectrometry (MALDI-TOF MS) adds to the identification alternatives. However, neither sophisticated equipment nor high technical expertise is an option in rural clinics in countries where *N. brasiliensis* is present. State economic resources for diagnosis are allocated to diseases with higher morbimortality than mycetoma, so only research institutes and high-specialty hospitals diagnose cases with great deformity or possible mortality. Finally, physicians opt to prescribe prolonged treatment with antibiotics such as trimethoprim-sulfamethoxazole, amoxicillin, and amikacin (Boiron and Stynen, 1992; Pujic et al., 2015; Develoux, 2016; Cuadro Básico y Catálogo de Medicamentos, 2017; Bakhiet et al., 2018; Verma and Jha, 2018; Vera, 2021). In Mexico, the National Epidemiological Surveillance System (SINAVE) does not consider nocardiosis a national threat owing to its low morbidity; however, its ubiquity in nature and its capacity to act as an intracellular pathogen should be taken into account, and if a quick and accurate diagnosis can be made, epidemiological information would surely change. Fourier transform infrared (FT-IR) technology is a tool used to study and identify chemical substances or functional groups to characterize new materials or to identify and verify known and unknown samples. It is a fast, non-destructive, simple, inexpensive, and high-performance analytical tool based on the differential vibrational modes of the chemical bonds of different functional groups when exposed to an infrared beam (Mendelsohn, 2007). FT-IR technology has been used to identify and type bacteria clones such as *E. coli*, *Klebsiella pneumoniae*, and *Acinetobacter baumannii*, among others. Because the fingerprint region (900–400 cm^–1^) has high variability, a visible colony result with high repeatability and reliability can be obtained in a single day and very quickly (Novais et al., 2019; Xie et al., 2022). Some actinomycetes have already been identified and typed by FT-IR, such as *Arthrobacter*, *Brevibacterium*, *Corynebacterium*, *Micrococcus*, *Rhodococcus*, *Mycobacterium bovis*, and other non-tuberculous mycobacteria (Amiel et al., 1997; Oberreuter et al., 2002; Winder et al., 2006). However, there are no reports of *N. brasiliensis* being identified or typed by FT-IR, so our work intends to provide the spectrum.

**FIGURE 1:**
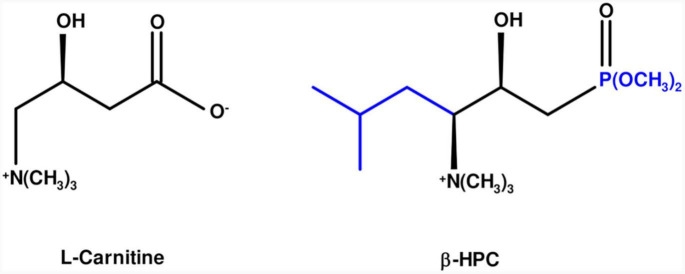
Molecular comparison of L-carnitine and β-HPC. On the left, in the black line structure, L-carnitine is shown as an amino acid derivative with seven carbons, a carboxylate, and a quaternary amino group. On the right, the additions and changes to β-hydroxyphosphocarnitine or β-hydroxy-γ-aminophosphonate are shown in blue: five carbons, a phosphonate in carbon one and an isopropyl group in carbon 4.

L-carnitine is an amino acid derivative; it has a chain of four carbon atoms, including a carboxylate, a hydroxyl group, and a quaternary amine with three methyl groups. On the other hand, the β-hydroxy-γ-aminophosphonate (β-HPC) has a chain of seven carbon atoms and a phosphonate group (two methyl groups), a hydroxyl group, a quaternary amine with three methyl groups. L carnitine (Figure 1). This modification confers greater solubility, stability, and potency (Kaboudin et al., 2022). β-HPC was proposed for the treatment of metabolic syndrome because, in animal models, it reduced blood levels of insulin, triglycerides and cholesterol in the liver and serum (Gómez-Solís et al., 2011; Reyes-Esparza et al., 2014; De la Cruz-Cordero et al., 2016). Similarly, it decreased steatosis and obesity in obese Zucker *fa/fa* rats and increased the concentration of CD3^+^/CD4^+^ lymphocytes (Reyes-Esparza et al., 2013; Sánchez-Quevedo et al., 2020). *N. brasiliensis* is identified by its hydrolytic activity on compounds of a protein and lipid nature. Biochemical tests such as hydrolysis of casein, L-tyrosine, egg yolk, and Tween 80 give a simple positive or negative reaction on the surface of agars. However, it is necessary to know the remaining functional groups on a substrate hydrolyzed by *N. brasiliensis* and to verify that β-HPC affects the hydrolytic activity of the bacterium when these previously identified functional groups disappear or are modified. If so, the bacterium plus analog can be proposed as a biotechnological tool since it could increase the production of metabolites or increase hydrolysis on substrates that could be contaminants or harmful in various productive processes (Engelbrecht et al., 2021). The FT-IR technique in microbiological applications has been proposed since the 1990s (Helm et al., 1991; Naumann et al., 1991) and is currently employed in the discrimination, classification, and identification of bacteria as a fast, inexpensive, and high-throughput method (Novais et al., 2019; Rodrigues et al., 2020; Passaris et al., 2022). Other interesting applications of the FT-IR technique include the evaluation of physiological responses to stress (Toziou et al., 2018), the measurement of host–pathogen interactions (Grunert et al., 2014), and the detection of bacterial metabolites (Cox et al., 1978). Therefore, in this work, we used FT-IR to demonstrate the effect of β-HPC on the catabolism of *N. brasiliensis* on the different representative substrates mentioned above. We found changes in the signals of the characteristic functional groups of each substrate and, in some cases, the absence of signals, mainly in tyrosine hydrolysis, resulting in a specific infrared spectrum for each sample. With the results, we demonstrated that β-HPC could be used as an additive to increase bacterial metabolism, and these findings were reinforced by statistical analysis.

## 2. Materials and methods

### 2.1. Bacterial strain

The *N. brasiliensis* strain HUJEG-1 (ATCC 700358) was used exclusively as a biochemical purity control of the experimental strain. The FM-825 strain was isolated from a patient with mycetoma and was kindly donated by Dr. Francisca Hernandez from the Faculty of Medicine of the National Autonomous University of Mexico (UNAM); it had previously been identified with morphological, biochemical, and molecular procedures and was used for all experiments. The FM-825 was grown in brain and heart infusion broth (BHI; Dibico, Cuautitlán Izcalli, Mexico) at 26°C for 5–7 days according to the previously observed (Conde et al., 1982). The colonies of *N. brasiliensis* grew on the surface of the BHI broth, forming a compact film, which was macerated in a Tenbroeck tissue grinder. A suspension was adjusted to 1.5 × 10^8^ CFU/ml McFarland standard in peptone water (0–73 g/mL; Fluka Analytical, Sigma-Aldrich, USA).

### 2.2. Preparation of culture media

The media preparation instructions were followed according to the López-Martínez and Cowan and Steele procedures (Barrow and Feltham, 1993; López-Martínez et al., 2013) with slight modifications. To evaluate the hydrolysis of casein: 0.1 g of skim milk (Difco, BD^®^Cat. No. 232100) was dissolved in 10 mL of distilled water and sterilized by heating at 110°C for 10 min. It was stored at 4°C before use. A total of 0.2 g of skim milk was used for the calibration curve and twofold dilutions were made in 2 mL of pyrogen-free sterile water (Pisa, Mexico). The concentration is shown in Table 1. To evaluate the hydrolysis of L-tyrosine: 0.23 g of nutrient broth (Difco, BD^®^ sup. No. 234000), 0.2 g of 4-Hydroxyphenylalanine, 0.05 g of Tyr (T4321 Millipore-Sigma^®^) were solubilized in 10 mL of distilled water at 25°C (Pubchem 6047) and sterilized at 115°C for 15 min. The broth was kept at 25°C. For the calibration curve, 0.2 g of l-tyrosine was used, and twofold dilutions were made in 2 mL aliquots of pyrogen-free sterile water aliquots (Pisa, Mexico). The concentration is shown in Table 1.

**TABLE 1:** Resulting in concentrations from twofold dilutions of substrates contained in 2 mL.

Dilution of substrates	1	2	3	4	5
Egg yolk (μL)	1500	750	**375**	187.5	93.75
Tween 80%	2	**1**	0.5	0.25	0.13
Casein (g)	20	**10**	5	2.5	1.25
L-tyrosine (g)	20	10	**5**	2.5	1.25

The bacteria inoculum 1.5 × 10^8^/mL was seeded. The highlighted squares are the usual concentrations per liter from the Cowan and Steel and López-Martínez manuals to reveal *N. brasiliensis* substrate hydrolysis. Bold values represent the concentrations regularly used to detect the hydrolytic activity of any bacteria. The dilutions are part of the calibration curve used.

To evaluate lipase activity: Tween 80 medium was prepared and 0.1 g of Casein peptone (Dibco), 0.05 g NaCl, 0.001 g CaCl_2_: 2H_2_O was suspended in 10 mL distilled water and sterilized at 121°C for 15 min. Tween 80 was sterilized at the same parameters and 0.1 mL was added aseptically to the flask to give a final concentration of 1%. The broth was maintained at 4°C before use. A total of 0.2 mL was used for the calibration curve and twofold dilutions were made in 2 mL aliquots of pyrogen-free sterile water (Pisa, Mexico). The concentration is shown in Table 1. Egg yolk broth base: 4 g of casein peptone, 0.5 g Na_2_HPO_4_, 0.2 g NaCl, a 0.5% solution and 0.2 g glucose. Subsequently, 20 μL of a MgSO_4_-7H_2_O solution was added to 10 mL of distilled water and sterilized at 121°C. One or two white eggs were disinfected on their surface; the yolks were separated from the whites in a sterile environment and under aseptic conditions, 750 μL was poured into the sterilized solution. For the calibration curve, 1500 μL of yolk was added to the sterilized solution and twofold dilutions were made in 2 mL aliquots of pyrogen-free sterile water (Pisa, Mexico). The concentration is shown in Table 1. The calibration curves for each substrate were made from five dilutions, as shown in Table 1, to compare and quantify the remaining substrates as products of the hydrolysis of *N. brasiliensis* and *N. brasiliensis* + β-HPC.

### 2.3. Preparation of β-hydroxy-γ-aminophosphonate (β-HPC) solution

Dr. Jorge Alberto Reyes Esparza from the Autonomous University of Morelos (UAEMor) kindly donated the experimental compound (β-HPC). A total of 64 μg/mL was solubilized in distilled water and filtered with a 33 mm diameter sterile syringe filter with a 0.22 μm pore size hydrophilic PVDF membrane (Millipore, USA). The β-HPC solution was added to the medium for hydrolysis evaluation.

### 2.4. Procedure for induction of hydrolysis

In 24-well clear flat bottom polystyrene microplates, sterile gamma irradiated (Nunclon Δ, Thermo Science, USA), 1800 μL of substrates per well were poured, plus 100 μL of β-HPC in triplicate. From a 1.5 × 10^8^ suspension of *N. brasiliensis*, a dilution was made to a work concentration (1 × 10^5^), and 100 μL was seeded in each well. In control hydrolysis wells, the bacterium inoculum was not seeded. The microplates were incubated at 25°C for 7 days in a stirring orbital plate.

At the end of the 7 days, the bacterium was separated from the medium using three centrifugation cycles (5000 g/10 min) each. Bacterial broth was defined as *N. brasiliensis*. The broth of bacteria and β-HPC was defined as *N. brasiliensis* + β-HPC.

An inoculum of *N. brasiliensis* was seeded in BHI broth. At the end of the 7 days, the bacterium was separated from the medium using three centrifugation cycles (5000 g/10 min) each. The pellet was extended in glass slides and heated at 45°C.

### 2.5. Preparation of samples for the FT-IR procedure

From each dilution for the calibration curve of each substrate, a sample of 300 μL was placed on the surface of a new and clean glass slide and heated at 45°C until the humidity was absent. From triplicates of the hydrolysis experiment, 300 μL was taken and heated at 45°C on new and clean glass slides.

### 2.6. FT-IR spectra specifications

Spectra were acquired with the Cary 630 benchtop FT-IR spectrometer (Agilent, 5301 Stevens Creek Blvd., Santa Clara, CA, USA) with a sample module of diamond attenuated total reflection (ATR). The absorbance spectra were collected between 4000 and 400 cm^–1^ at a spectral resolution of 4 cm^–1^ with 50 scans co-added and averaged and 50 background scans.

The relevant peaks were obtained with the Spectragryph v1.2.15 optical spectroscopy software, SciDAVis 2.4.0, and Python 3.8.8 software.

### 2.7. Statistical analyzes

Data obtained from bacterium hydrolysis with β-HPC and without β-HPC were interpolated with data obtained from the calibration curve to compare and obtain the quantity of hydrolyzate. Linear regression analysis, least squares analysis, statistical summaries, and principal component analysis (PCA) were performed in R Commander 2.5–1, and GraphPad Prism 5 was used to obtain graphics.

## 3. Results

Infrared spectra between 1400 and 400 cm^–1^ (fingerprint region with the highest variability between the experimental groups) of *N. brasiliensis* and *N. brasiliensis* + β-HPC corresponding to the 7 days of culture were analyzed.

### 3.1. Differences in hydrolyzed substrates between experimental conditions by FT-IR

**FIGURE 2:**
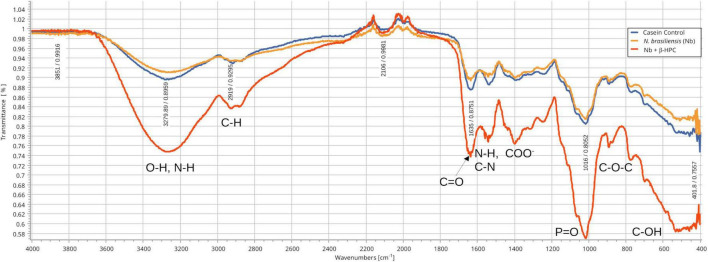
Comparison of FT-IR spectra of the hydrolytic activity of *N. brasiliensis* on casein with and without β-HFC. Culture medium alone, control (blue line), culture medium with 7 days of growth of *N. brasiliensis* (orange line), and culture medium with 7 days of *N. brasiliensis* + β-HPC (red line); the increase in the signals of the alcohol functional group (O–H), alkanes (C–H), amides I, II, and III (*C* = O, N-H, C-N), the carboxylic acid (COO–), phosphate (*P* = O), and ethers (C–O–C) characteristic of amino acids, when *N. brasiliensis* was incubated in the presence of β-HPC indicating greater hydrolysis of casein.

**FIGURE 3:**
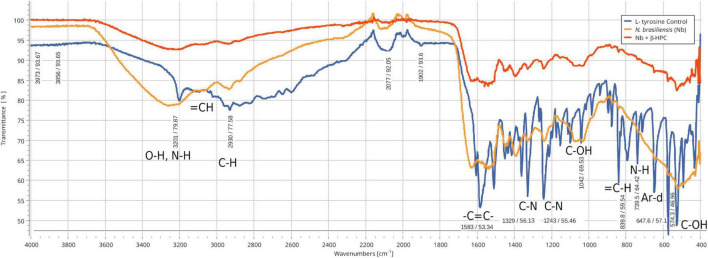
Comparison of FT-IR spectra of the hydrolytic activity of *N. brasiliensis* on L-tyrosine with and without β-HFC. Culture medium alone, control (blue line), culture medium with 7 days of growth of *N. brasiliensis* (orange line), culture medium with 7 days of *N. brasiliensis* growth + β-HPC (red line). A decrease in the bands corresponding to the functional groups of L-tyrosine is observed with respect to the control when *N. brasiliensis* is incubated in the culture and the effect is more marked when *N. brasiliensis* + β-HPC is added since the characteristic signals of this amino acid that correspond to the functional group (OH), to the amino acid (NH), to alkenes (*C* = C), and to the aromatic signals (Ar d) notoriously decrease, these signals show a transmittance between 85 and 95 cm^–1^ (red line).

**FIGURE 4:**
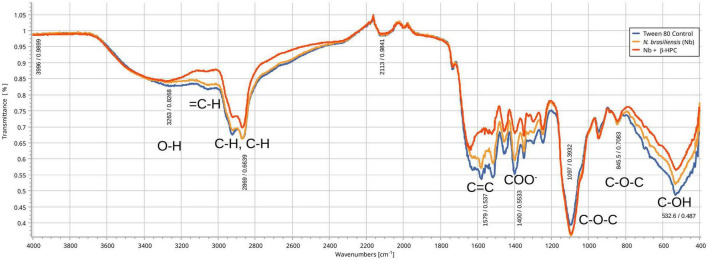
Comparison of FT-IR spectra of the hydrolytic activity of *N. brasiliensis* on tween 80 with and without β-HFC. Culture medium (control blue line), hydrolyzate of 7 days of *N. brasiliensis* growth (orange line), hydrolyzate of growth of 7 days of *N. brasiliensis* + β-HPC (red line). A decrease in the alcohol group (O–H) is observed, as well as the signals of alkenes (= C-H), alkanes (C–H), and carboxylic acids (COO). In contrast, more intense signals are detected mainly from ethers (C–O–C), both groups of signals, when accumulating and intensifying the signal, also indicate an increase in the utilization of Tween 80 when *N. brasiliensis* + β-HPC is incubated.

**FIGURE 5:**
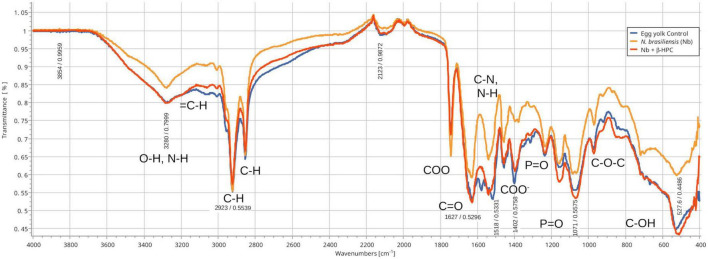
Comparison of FT-IR spectra of the hydrolytic activity of *N. brasiliensis* on egg yolk with and without β-HFC. Culture medium alone, control (blue line), culture medium with 7 days of *N. brasiliensis* growth (orange line), culture medium with 7 days of *N. brasiliensis* growth + β-HPC (red line). A decrease in alcohol (O–H) signals is observed, as well as alkenes (= C-H, C = C), alkanes (CH_3_, C-H), and signals of the COO ester. In contrast, C-C and C-O-C had strong signals. At 3280 cm^–1^, O-H, and N-H stretches were detected. At 2923 cm^–1^, C-H strain signals were detected with C sp^3^ hybridization (absorbance at all three: 0.55), and at 2850 cm^–1^, C-H was observed (absorbance at 3 0.64). At 1750 cm^–1^ COO^–^ signals characteristic of esters were detected (0.71, 0.65, 0.71); amide I carbonyl (*C* = O) signal was detected at 1627 cm^–1^; amide II N-H, C-N at 1518 cm^–1^; a signal characteristic of carboxylic acids (COO–) at 1400 cm^–1^; and *P* = O at 1250 cm^–1^. A strong signal (phosphate *P* = O) was identified at 1071 cm^–1^. At 950 cm^–1^, there was a signal of alcohol (C–OH); beyond the fingerprint zone, there was also a robust C-OH signal at 550 cm^–1^.

From the hydrolysis comparison, the figures show three overlapped lines: the blue line represents the unseeded substrate (control), the orange line is the resulting spectrum of a substrate already hydrolyzed by *N. brasiliensis*, and the red line is the hydrolyzate of *N. brasiliensis* plus β-HPC (*N. brasiliensis* + β-HPC) during the seven experimental days. Transmittances (**T**) were determined using the relevant peaks of Spectragryph^®^ enclosed in parentheses: control, hydrolysis of *N. brasiliensis*, hydrolysis of *N. brasiliensis* + β-HPC. The **casein** broth (Figure 2) showed a broad signal at 3279 cm^–1^ corresponding to stretches (OH), probably serine and threonine. NH_2_ stretches (T: 0.9, 0.91, 0.75 of control, hydrolysis of *N. brasiliensis*, hydrolysis of *N. brasiliensis* + β-HPC, respectively) were also observed. At 2919 cm^–1^ (T: 0.94, 0.94, 0.84), C-H signals with sp^3^ hybridization were found. Two signals corresponding to amides I and II were detected and secondary amide stretches (T: 0.904, 0.88, 0.78). At 1635 cm^–1^, we detected *C* = O amide I of the peptide bond (T: 0.88, 0.89, 0.74). At 1550 cm^–1^, N-H flexion and C-N stretch (T: 0.88, 0.90, 0.78) were found. A transmittance signal was detected at 1400 cm^–1^ corresponding to *C* = O belonging to the carboxylic acid COO (T: 0.89, 0.89, 0.76). Additionally, a signal appeared at 1016 cm^–1^ corresponding to the phosphate signal (T: 0.81, 0.81, 0.58). Inside and further beyond the fingerprint zone, C-O-C bending tension signals (T: 0.89, 0.89, 0.76) were observed at 850 cm^–1^, and at 550 cm^–1^, C-OH signals (T: 0.78, 0.81, 0.58) were detected. In the **L-tyrosine** spectrum (Figure 3), the relevant peaks were represented in the substrate (blue line); they were compared with the bacterial hydrolysis. The red line representing *N. brasiliensis* + β-HPC had the highest transmittance, which means that it was completely hydrolyzed. *N. brasiliensis* had shifts and shortenings in its peaks, especially in the fingerprint area. At 3270 cm^–1^, there was a broad stretching corresponding to OH (T: 0.85, 0.78, 0.92), and stretch signals were found at 3201 cm^–1^ corresponding to NH from amide (T: 0.80, 0.78, 0.93). At 2930 cm^–1^, asymmetric stretching C-H shifts (T 0.76, 0.83, 0.94) were found. *C* = O signals from amide I were found at 1650 cm^–1^ (T: 0.62, 0.64, 0.85). At 1583 cm^–1^, there was stretching -*C* = C- (T: 0.53, 0.63, 0.84). A characteristic signal of L-tyrosine located at 1515 cm^–1^ was found (T: 0.57, 0.62, 0.84). At 1329 cm^–1^, stretches belonging to aromatic C-N (T: 0.56, 0.70, 0.88) were found within the mixed region. At 1243 cm^–1^, we detected C-N (T: 0.55, 0.69, 0.87). At 1200 cm^–1^ within the mixed region were detected CO and C-O-H (T: 0.70, 0.74, 0.90). At 839 cm^–1^, bending = C-H stretches were detected belonging to the whole L-tyrosine (T: 0.59, 0.78, 0.93). At 739 cm^–1^ there were N-H torsional vibrations (T: 0.64 0.73, 0.89). At 647 cm^–1^, Ar-d (aromatic) (T: 0.64, 0.72, and 0.88) was detected. Finally, at 550 cm^–1^, there were bendings of C-OH (T: 0.48, 0.58, and 0.82). Another substrate was **Tween 80** (Figure 4), which allowed the growth of *N. brasiliensis*; the spectra were discrete at 3263 cm^–1^ where the O-H group was present (T: 0.83, 0.82, 0.84). At 3030 cm^–1^, = C-H signals (T: 0.81, 0.84, 0.87) were found. At 2955 cm^–1^, there were vibrations and C-H stretching (T: 0.68, 0.69, 0.74), and at 2869 cm^–1^, there were C-H signals (T: 0.66, 0.66, 0.69). *C* = C signals were found at 1620 cm^–1^ (T: 0.56, 0.60, 0.62). At 1579 cm^–1^, an intense band of *C* = C groups was detected (T: 0.53, 0.58, 0.68). At 1400 cm^–1^, a band corresponding to the *C* = O from of COO^–^ symmetric stretching was detected (T: 0.56, 0.60, 0.68). All these substrate signals decreased, although slightly, when β-HPC was added to *N. brasiliensis*. At 1100 cm^–1^, there was a pronounced peak representing C-O-C deformations (T: 0.49, 0.35, 0.35), while at 845 cm^–1^, a C-O-C stretching (T: 0.72, 0.72, 0.72) was detected. Finally, at 532 cm^–1^, a strong and broad signal of Tween 80 was detected giving a clear C-OH signal (T: 0.48, 0.54, 0.56) was detected in the substrate, which is decreased by hydrolysis by *N. brasiliensis* + β-HPC. The **egg yolk** was considered only as a lipid substrate; however, we found functional groups characteristic of amino acids. The FT-IR spectrum detected that the lines of the experiments overlapped (Figure 5); therefore, the substrate spectrum was observed to run parallel with *N. brasiliensis* + β-HPC. At 3280 cm^–1^, signals corresponding to O-H, N-H stretches (T: 0.80, 0.84, 0.80) were observed. At 3020 cm^–1^, = C-H signals were observed (T: 0.82, 0.88, 0.84). At 2923 cm^–1^, C-H strain signals with C sp^3^ hybridization were detected (T: 0.55, 0.55, 0.56), and at 2850 cm^–1^ C-H (T: 0.60, 0.65 0.66) were found. At 1750 cm^–1^, the characteristic COO signals of esters were identified (0.71, 0.65, 0.71). Amide I *C* = O signals were present at 1627 cm^–1^ (T: 0.53, 0.59, 0.53). Amide II flexion N-H and stretching from C-N at 1518 cm^–1^ (T: 0.53, 0.64, 0.55) was detected. At 1402 cm^–1^, a characteristic signal of *C* = O from COO^–^ (0.57, 0.75, 0.62) was observed. At 1250 cm^–1^, *P* = O was identified (T: 0.65, 0.66, 0.65); similarly, there was a strong phosphate signal (*P* = O) at 1071 cm^–1^ (T: 0.56, 0.60, 0.54) the transmittance of this signal decreased when β-HPC was added to *N. brasiliensis*. At 950 cm^–1^, there was a bending out of the plane with a C-OH signal (T: 0.66, 0.74, 0.66). Beyond the fingerprint zone, there was also a strong C-OH signal at 515 cm^–1^ (T: 0.45, 0.60, 0.42) that also decreased when β-HPC was added to *N. brasiliensis*.

### 3.2. Findings of relevant peaks

**FIGURE 6:**
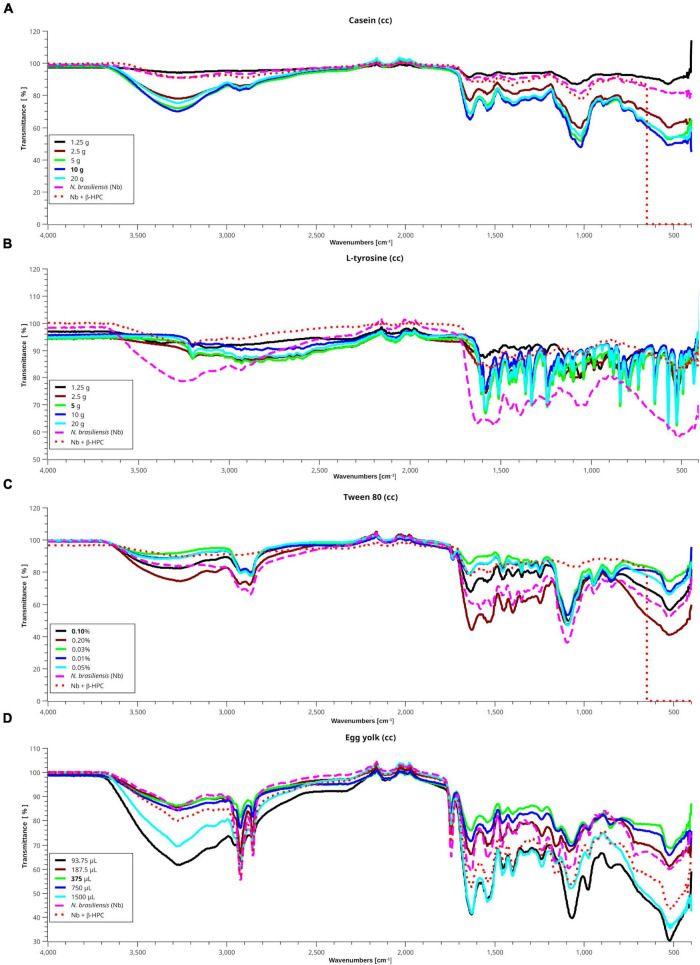
Interpolation of the hydrolysis of *N. brasiliensis* on substrates within the calibration curve. In the presence and absence of β-HPC with different standard concentrations from the calibration curves (cc) of each substrate, using the SCiDAVis program. **(A)** Casein spectra (cc), a transmittance value tending to 100 indicates degradation of this substrate. The spectra of hydrolyzed casein (10 g) with *N. brasiliensis* alone (dashed fuchsia line) or *N. brasiliensis* + β-HPC (dotted red line) are similar to that of 1.25 g (black line), proving that hydrolysis took place. **(B)** The L-tyrosine spectra showed the functional groups previously described between 1500 and 400 cm^–1^; even when the detected signals are shorter due to dilution. The spectrum of hydrolyzed L-tyrosine by *N. brasiliensis* (dashed fuchsia line) showed a broader OH signal at 3650 cm^–1^ with a transmittance value of 75, and at 1550 cm^–1^, with a transmittance of 60. On the contrary, the spectrum of hydrolyzate by *N. brasiliensis* + β-HPC is similar to that of 1.25 g (black line) with a transmittance greater than 90, indicating almost complete hydrolysis of the substrate. **(C)** In the Tween 80 cc, the hydrolyzate by *N. brasiliensis* corresponds to that of 0.2% concentration of this substrate. However, such results indicated a higher concentration of hydrolyzed compounds, since the concentration for the identification of this substrate is 0.1%; the spectrum hydrolyzed by *N. brasiliensis* + β-HPC (red dotted line) resembles that of 0.025% (green line). **(D)** Signals in egg yolk cc spectra show higher amplitude when this substrate is diluted, and in the 3000–3500 cm^–1^ range, the hydrolyzed product by *N, brasiliensis* resembles that of 375 μL (green line) with a transmittance value of 86. The hydrolyzed lipid of *N. brasiliensis* + β-HPC correlates to that of 750 μL (deep blue line) with a transmittance value of 82. *N. brasiliensis* + β-HPC were closest to the highest concentrations. In the protein window (1800–1500 cm^–1)^, the substrate hydrolyzed by *N. brasiliensis* has a transmittance value of 60, and that hydrolyzed by *N. brasiliensis* + β-HPC presented a transmittance value of 54; such differences were similar throughout the mix and polysaccharide windows. However, broad signals were observed at 93, 75, and 1500 μL with transmittance values of 30 and 38 in the fingerprint area. In the presence of β-HPC, a signal with a transmittance value signal of 44 was detected.

Spectragryph open source software was chosen to obtain the differences between the peaks of the substrate curve, and the SciDAVis 2.4.0 free application for scientific data analysis and visualization was used to obtain the stacked spectra of the calibration curves; such images also included the spectra obtained from the hydrolysis of *N. brasiliensis* and *N. brasiliensis* + β-HPC (Figure 6). After five dilutions, the same peaks remained but softened, and transmittance approached 100. *N. brasiliensis* distinguished itself among the other species of hydrolyzing **casein**. As expected, the spectra of *N. brasiliensis* (Figure 6A) (fuchsia dashed line) and *N. brasiliensis* + β-HPC (dotted red line) were paralleled by the casein concentration of 1.25 g (black line). Only a difference occurred at 550 cm^–1^ when the substrate containing β-HPC was exhausted.

The calibration curve for **L-tyrosine** (Figure 6B) calibration curve indicated how the concentration faded out; however, the hydrolyzate of *N. brasiliensis* (fuchsia dashed line) showed a slight displacement in 3270 cm^–1^ of a broad OH signal with a transmittance of 78 vs. 92 of β-HPC (dotted red line). This substrate increased to 100; unlike the other calibration curve spectral reads, the one with β-HPC disappeared entirely from the spectrum. From 3200 to 1500 cm^–1^, where the *C* = C- and *C* = O were present, *N. brasiliensis* once again had a stronger and broader signal transmittance of 63 vs. 84 of *N. brasiliensis* + β-HPC at 550 cm^–1^. *N. brasiliensis* showed 58 transmittance v. 82 from *N. brasiliensis* + β-HPC. *N. brasiliensis* equalized the 20 g concentration curve. On the contrary, *N. brasiliensis* + β-HPC equalized the 1.25 g curve.

In the **Tween 80** calibration curve (Figure 6C), spectra were seen at 3263 cm^–1^ in a wide OH in 0.2% were seen; *N. brasiliensis* transmittance was 82 while *N. brasiliensis* + β-HPC transmittance was 84. At 2955 and 2869 cm^–1^, the two characteristic peaks of Tween 80 were present (Tween 80, 2022), although with shortenings. *N. brasiliensis* had 62 transmittance compared to 85 from *N. brasiliensis* + β-HPC. From 1620 to 1180 cm^–1^, *N. brasiliensis* had a transmittance value of 60. In contrast, *N. brasiliensis* + β-HPC had a transmittance of 82. At 1100 cm^–1^, *N. brasiliensis* showed a 38 transmittance value, far below the 82-transmittance value of *N. brasiliensis* + β-HPC; beyond 555 cm^–1^, the tween 80 was entirely consumed by *N. brasiliensis* + β-HPC, and at 550 cm^–1^, *N. brasiliensis* remained at a 58 transmittance value.

The **egg yolk** is a complex suspension (Parkinson, 1966; Kononov et al., 1988; Molina, 2020; Xiao et al., 2020) whose content of organic acids such as oleic acid is also present in Tween 80. The lipids, proteins, and minerals were metabolized in a different way: 93.75 μL (black line) was the highest dilution with the lowest transmittance signal, even the peaks were smooth and heavy, mostly in the fingerprint zone; this highly diluted substrate was 1500 μL, the highest concentration. In (Figure 6D), from 3500 to 1650 cm^–1^, the experimental hydrolyzates were close to 375 μL. Transmittance and concentration at 3300 cm^–1^ from stretches of O-H, N-H showed a signal from *N. brasiliensis* with a transmittance of 84, while *N. brasiliensis* + β-HPC had a transmittance of 80. At 2923 cm^–1^, CH_2_ was asymmetrical, with a transmittance value of 54, corresponding to both experimental Nocardia species. At 2850 cm^–1^, the signal corresponding to symmetric CH_2_ showed another peak with a transmittance value of 65, which was also reached at the exact location and height of *N. brasiliensis*, and a transmittance value of 66 was reached by *N. brasiliensis* + β-HPC. The same was observed at 1750 cm^–1^ with a transmittance of 65 for *N. brasiliensis* and 71 corresponding to *N. brasiliensis* + β-HPC. At 1627 cm^–1^, the transmittance corresponding to *N. brasiliensis* + β-HPC was 53, and Nocardia alone was 59. At 1518, 1402, 1250, and 1071 cm^–1^, *N. brasiliensis* showed transmittance values of 64, 67, 66, and 54, respectively, and *N. brasiliensis* + β-HPC rose in transmittance at 55, 56, 65, and 54, respectively. At 1071 cm^–1,^ a transmittance of 54 from *N. brasiliensis* + β-HPC contrasted with 60 for *N. brasiliensis*. Finally, the fingerprint zone at 515 cm^–1^ showed strong signals from 1500 to 93.75 μL with transmittances of 30 and 38, followed by *N. brasiliensis* + β-HPC with a transmittance of 42. *N. brasiliensis* alone had a transmittance of 60. Hydrolysis of *N. brasiliensis* approached 187.5 μL concentration, and *N. brasiliensis* + β-HPC approached 1500 μL from 1600 to 450 cm^–1^.

### 3.3. Linear regression analysis

**FIGURE 7:**
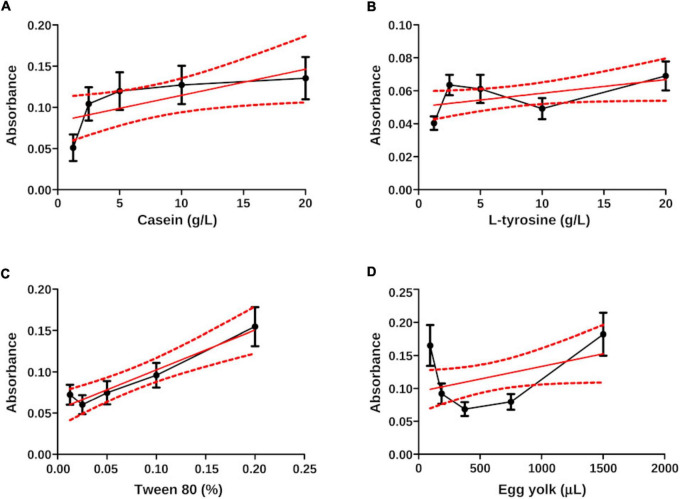
Linear regression plots (x, y) of the interpolated data of *N. brasiliensis* on the calibration curve. **(A)** Casein did not show a linear relation even when the values were within the control limits (dotted lines in red). The *r*^2^ value was 0.70. **(B)** The zigzagging behavior in L-tyrosine did not show linear regression, *r*^2^ was 0.67. **(C)** The first plotted data prevented by Tween 80 showed a linear regression also affected by correlation, the r^2^ was 0.69. **(D)** The curve relation in egg yolk exceeded the control limits, the *r*^2^ was 0.73. The plots were generated in GraphPad Prism 5.0.

After the calibration curve, the dilutions were read in the Spectragryph software and the transmittance data of each broth were analyzed in the R Commander software. As expected, the relation between concentration (***x***) and transmittance (***y***) was not linear. The *r*^2^ value of the **casein** broth was 0.7, **L-tyrosine** 0.67, Tween **80** 0.69, and **egg yolk** 0.73. The low strength of the Pearson correlation coefficient contrasted with the *P*-value, since linear regression showed statistical differences (Table 2 and Figure 7). The purpose of performing a calibration curve was to interpolate the concentrations of *N. brasiliensis* and *N. brasiliensis* + β-HPC in every substrate after hydrolysis to corroborate the differences and concentration changes observed in FT-IR spectra at the end of the experimentation.

**TABLE 2:** Comparisons of hydrolyzates resulting from catabolism between *N. brasiliensis* and *N. brasiliensis* + β-HPC and within a calibration curve from casein, L-tyrosine, Tween 80, and egg yolk.

Comparison of transmittances and statistics of hydrolyzates inside calibration curve					
Casein	cm^–1^	Statistics	*N. brasiliensis* (∞)	*N. brasiliensis* + β-HPC (¥)	Particularities
	3279		91	75	1.25 g ∞¥ *r*^2^ 0.70/*P* = 0.02637
	2919		94	84	
	1550		90	78	
	1635		89	74	
	1400		89	76	
	1016		81	58	
	850		89	76	
	550		81	58	
		Linear regression	1.25 g	5 g	
		Least-squares	10 g	10 g	*r*^2^ 1.0/*P* < 0.0001*** ∞**^–^**¥
		Summaries	1.25 g	5 g	Shapiro–Wilk normality test *p* = 0.05 0.00000000003024
		APC (eigenvalues)	0.393163	0.3008444	0.3936127/20 g
L-tyrosine	3270		78	92	20 g∞, 1.25 g ¥
	3201		78	93	
	2930		83	94	
	1583		63	84	
	1515		62	84	
	1329		70	88	
	1243		69	87	
	1200		74	96	
	839		78	93	
	739		73	89	
	647		72	88	
	530		58	82	
		Linear regression	2.5	2.5	*r*^2^ 0.67/*P* = 0.000001983***
		Least-squares	5 g	5 g	*r*^2^ 0.67/*P* = 0.0041**
		Summaries	20 g	10 g	Shapiro–Wilk normality test *p* = 0.05 5.03E-14
		APC (eigenvalues)	0.3663484	0.3735338	0.3782673/20 g∞
Tween 80	3263		82	84	0.2%∞, 0.03% ¥
	3030		84	87	
	2920		69	74	
	2869		66	69	
	1620		60	62	
	1579		58	68	
	1400		60	68	
	1100		35	35	
	845		72	72	
	532		54	56	
		Linear regression	0.01%	0.01%	*r*^2^ 0.69/*P* = 0.0001683***
		Least-squares	1.00%	1.00%	*r*^2^ 1.0/*P* < 0.0001***
		Summaries	2.00%	2.00%	Shapiro–Wilk normality test *p* = 0.05 9.07E-10
		APC (eigenvalues)	0.3959531	0.2651606	0.3946757/0.0125%∞
Egg yolk	3280		84	80	1500 μL∞, 187.5 μL ¥
	3020		88	84	
	2923		55	56	
	2850		65	66	
	1750		65	71	
	1627		59	53	
	1518		64	55	
	1402		75	62	
	1250		66	65	
	1071		60	54	
	950		74	66	
	515		60	42	
		Linear regression	1500 μL	375 μL	*r*^2^ 0.73/0.001502*
		Least-squares	375 μL	375 μL	*r*^2^ 1.0/*P* < 0.0001***
		Summaries	187 μL	93.75 μL	Shapiro–Wilk normality test *p* = 0.05 0.000000006991
		APC (eigenvalues)	0.3743214	0.3807756	0.3743118/93.75 μL∞
					0.3800296/187.5 μL ¥

The table summarizes the relevant peaks obtained in the Spectragryph^®^ software, with additional statistics: linear regression, least squares, statistical summaries, and analysis of principal components (APC). Particularities account for differences among hydrolyzates. *N. brasiliensis* is represented by the infinity symbol (∞), *N. brasiliensis* + β-HPC is represented by the yen symbol (¥).

*P < 0.05, **P < 0.01, and ***P < 0.001.

To find a best-fit linear regression, we analyzed the peaks obtained from Spectragryph© through a least-squares method because they can handle spectroscopic values containing transmittance values at hundreds to thousands of wavelengths (Ripa, 2002; Hecker et al., 2012; Astorga, 2014; Spínola, 2020). We use the following equation to find the slope in (***m***):


$$m=\Sigma \,\begin{array}{c} n\\ i=1 \end{array}\,x\,i\,\left(y\,i-\hat{y}\right)\vee \Sigma \,\begin{array}{c} n\\ i=1 \end{array}\,x\,i\,\left(x\,i-\hat{x}\right)$$

and to find the intercept (***b***):


$$b=\hat{y}-m\cdot \hat{x}$$

Finally, we obtain *m* and *b*; thus, the equation “y” was performed:


y=m⋅x+b

**FIGURE 8:**
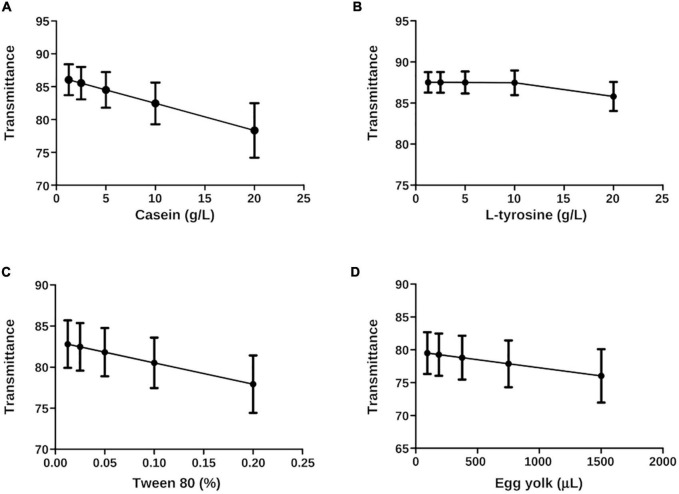
Least squares plots (x, y) of *N. brasiliensis* hydrolyzates interpolated on the calibration curve. The best fit was calculated. **(A)** Casein has linear regression and a pronounced slope with an *r*^2^ of 1.0. **(B)** L-tyrosine showed a feeble slope in the linear regression. Therefore, the *r*^2^ value was 0.67. **(C)** Tween 80 had linear regression, slope, and an *r*^2^ value of 1.0. **(D)** The egg yolk showed a linear regression. The slope was not as pronounced as the casein, and the *r*^2^ of Tween 80, was 1.0. The plots were generated in GraphPad Prism 5.0.

This statistical method allowed us to place the experimental *N. brasiliensis* and *N. brasiliensis* + β-HPC inside the calibration curve in the best fit using wavelength concentrations as (**x**) and transmittance values as (**y**). Figure 8 contrasts with Figure 7 in the sense that ordinary linear regression is not sufficient to find a relationship (linearity) and strength in the relationship (*r*^2^). The least squares method allows fitting the data to find a straight line and a strong correlation. The least squares method allows us to fit the data until we find the straight line and strong correlation, which we obtained. The interpolated calculated value was precisely fitted with the corresponding dilution value and the statistical figures ended as follows: from the calculated transmittance, casein reached an *r*^2^ value of 1.0, and both experimental Nocardia corresponded precisely to the dilution 1:2 (10 g). Concerning L-tyrosine, the transmittance of the calculated value *r*^2^ was 0.67; however, the regression lacked slope, although *N. brasiliensis* and *N. brasiliensis* + β-HPC corresponded to 1:3 dilution (5 g). In Tween 80, the experimental value of *r*^2^ was 1.0, and *N. brasiliensis* and *N. brasiliensis* + β-HPC corresponded to the second concentration (1%). Finally, the observed *r*^2^ calculated value of egg yolk was 1.0, and *N. brasiliensis* and *N. brasiliensis* + β-HPC fell to a 3:1 dilution (375 μL). The results of all the substrates of the calibration curve, as well as experimental hydrolyzates of *N. brasiliensis* and *N. brasiliensis* + β-HPC, also showed high statistical significance (*P* < 0.05) (Table 2) as expected, and the linear plots of the substrates were obtained, however, when fitting the values, the relevant numerical details provided by the increase in functional groups released by hydrolysis or their disappearance when consumed by *N. brasiliensis* are lost due to the best fit, so this model does not show significant differences between hydrolysis alone and that augmented by β-HPC.

### 3.4. Statistical summaries

**FIGURE 9:**
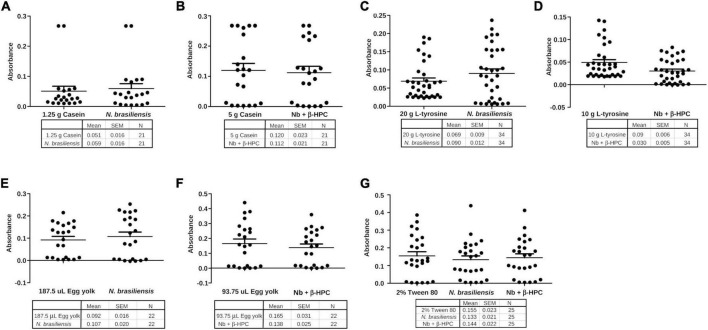
Grouping of data of the hydrolysis of *N. brasiliensis* within the calibration curve. **(A)** Hydrolysis of *N. brasiliensis* casein compared to calibration curve 1:16 dilution, corresponding to 1.25 g. **(B)** *N. brasiliensis* + β-HPC casein hydrolysis was close to the calibration curve 1:4 corresponding to 5 g. **(C)** The hydrolysis of *N. brasiliensis* on L-tyrosine approached the initial amount of the calibration curve (20 g). **(D)** *N. brasiliensis* + β-HPC hydrolysis approached a dilution of 1:2 of L-tyrosine from the calibration curve (10 g). **(E)** In egg yolk hydrolysis by *N. brasiliensis*, the dilution was 1: 8. **(F)** The hydrolysis of egg yolk from *N. brasiliensis* + β-HPC was greater because it approached the highest dilution. **(G)** *N. brasiliensis* and *N. brasiliensis* + β-HPC species over Tween 80 hydrolysis showed similarities to the 2% calibration curve; it represented the initial concentration to be diluted.

Differences were present among variances; we compared the hydrolyzate substrates with each other and the calibration curve through statistical summaries. The data obtained from FT-IR were subjected to a Shapiro–Wilk normality test and there was no normal distribution (Table 2). Central tendency measures such as mean and median were taken. Variance, standard deviation, and standard error were the dispersion measures used to visualize them in grouped plots. The metabolized **casein** of *N. brasiliensis* was almost the same at 1.25 g (1:16) as the calibration curve. While casein hydrolyzed by *N. brasiliensis* + β-HPC showed similarity to 5 g from the calibration curve (1:4) (Figures 9A, B). Regarding **L-tyrosine**, the statistical summaries obtained for this amino acid from *N. brasiliensis* approached 20 g of the calibration curve. Although L-tyrosine hydrolyzed by *N. brasiliensis* + β-HPC came close to 10 g (1:2) from the calibration curve (Figures 9C, D). Concerning **egg yolk** hydrolysis, when comparing *N. brasiliensis* and *N. brasiliensis* + β-HPC with the calibration curve, high hydrolysis took place: *N. brasiliensis* approached 187.5 μL (1:8), and the hydrolyzed substrate by *N. brasiliensis* + β-HPC was close to 93.75 μL (1:16) (Figures 9E, F). The surfactant Tween **80** was hydrolyzed similarly by *N. brasiliensis* and when it was fulled with β-HPC. The statistical metabolism numbers for *N. brasiliensis* and *N. brasiliensis* + β-HPC approached the calibration curve at 2% (initial concentration) (Figure 9G).

### 3.5. Principal component analysis

**FIGURE 10:**
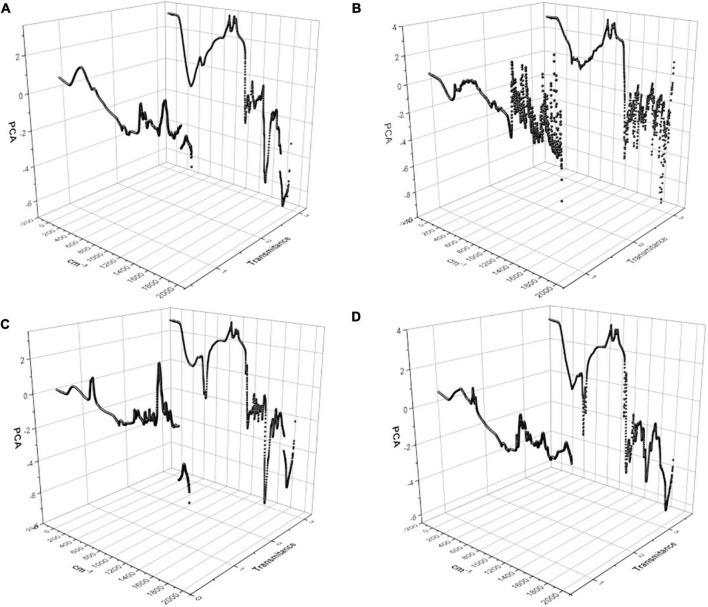
Principal component analysis (PCA) of the hydrolysis of *N. brasiliensis* within the calibration curve. Two main components were graphed that expressed the widest variation in the data set, a total of 1932 values given by FT-IR wavelength (X) and transmittance (Y). **(A)** Casein substrate at 1.25, 2.5, 5, 10, and 20 g, *N. brasiliensis* and *N. brasiliensis* + β-HPC. **(B)** L-Tyrosine substrate at 1.25, 2.5, 5, 10, and 20 g, *N. brasiliensis* and *N. brasiliensis* + β-HPC. **(C)** Tween 80 substrate at 0.1, 0.2, 0.025.0.01, and 0.5%, *N. brasiliensis* and *N. brasiliensis* + β-HPC. **(D)** Egg yolk substrate at 93.75, 187.5, 375, 750, 1500 mL, *N. brasiliensis* and *N. brasiliensis* + β-HPC. The PCA analysis was performed with OriginPro 2018 and R Commander 2.5–1.

As observed in Figure 6, differences between the hydrolyzate of *N. brasiliensis* and *N. brasiliensis* + β-HPC were present, and the number of variables detected by the wavelength of FT-IR (**x**) and the transmittance (**y**) was so large that it was necessary to narrow them over time. Thus, we consider a principal component analysis (PCA). The three-dimensional plots described two distinctive groups with high variability within the experimental groups, and the concentrations related to them were found in the calibration curves. They obtained seven PCs from a calibration curve and a hydrolyzed substrate. The first component was large enough to be analyzed. **Casein** (Figure 10A) had a 91% variance proportion and a variance of 6.38. The loading component value of the control FT-IR of 20 g was 0.3936127. The eigenvalue of *N. brasiliensis* hydrolyzate was 0.3931630, and the experimental *N. brasiliensis* + β-HPC eigenvalue was 0.3008444. The **L-tyrosine** had 85% proportion of variance also a component variance of 5.99, as observed in Figure 10B. The chosen eigenvalue of the control was 0.3782673, corresponding to 20 g from the calibration curve due to the closeness of the experiential value of *N. brasiliensis*, whose component load was 0.3663484, and *N. brasiliensis* + β-HPC, which showed 0.3735338. The first **Tween 80** PC had a proportional variance of 86% (Figure 10C) and a component variance of 6.06; the control eigenvalue of 0.3946757, corresponding to 0.01% of Tween 80 concentration after hydrolysis of *N. brasiliensis*, was close to the eigenvalue of 0.3959531, while the *N. brasiliensis* + β-HPC eigenvalue was 0.2651606. The first **egg yolk** PC showed a proportion variance of 97%. The component variance was 6.832437594 (Figure 10D); the control eigenvalue chosen from the calibration curve was 0.3743118, corresponding to 93.75 μL of egg yolk in the calibration curve. This value corresponded to the 0.3743214 eigenvalues of the hydrolyzate of *N. brasiliensis* hydrolyzate; this substrate gave us another control eigenvalue of 0.3800296 that corresponded to the 0.3807756 eigenvalues of *N. brasiliensis* + β-HPC corresponding to 187 μL.

### 3.6. FT-IR characterization of *N. brasiliensis*

**FIGURE 11:**
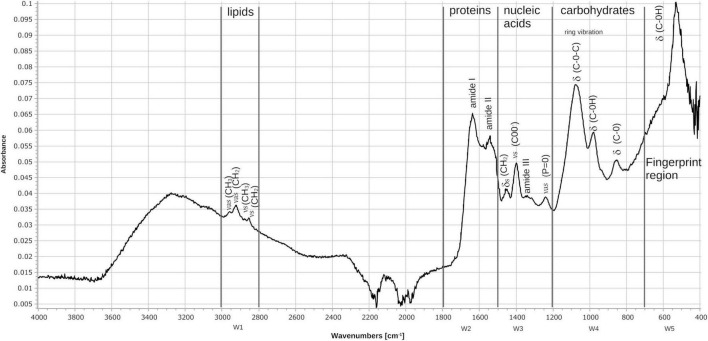
Fourier transform infrared (FT-IR) spectrum of the FM-825 strain of *N. brasiliensis* isolated from a mycetoma patient. The absorbance spectra were collected between 4000 and 400 cm^–1^ at a spectral resolution of 4 cm^–1^ with 50 coadded and averaged scans and 50 background scans. The typical spectrum of bacteria is observed showing the assignments of the main signals within the spectral windows (W1–W5). (v) stretching vibrations, (d) bending vibrations, (s) symmetric vibrations, (as) asymmetric vibrations.

The wavelengths commonly used by FT-IR for the characterization of bacteria are in the region of 4000 to 400 cm^–1^, which corresponds to the middle region of the infrared electromagnetic spectrum. The spectrum of *N. brasiliensis* (Figure 11), presented signals within the five spectral windows characteristics of bacteria (Helm et al., 1991; Naumann et al., 1991; Novais et al., 2019; Rodrigues et al., 2020; Jinghang et al., 2022; Passaris et al., 2022). Window 1 corresponds to lipids and ranges from 3000 to 2800 cm^–1^; for *N. brasiliensis*, the symmetric and asymmetric vibrations of the functional groups -CH_2_ and -CH_3_ of the fatty acids were detected. Window 2, which corresponds to proteins and peptides, ranges from 1800 to 1500 cm^–1^. The characteristics of amides I and II were notable in *N. brasiliensis*; specifically, signals were detected at 1635 cm^–1^ of amide I and 1550 cm^–1^ of amide II. Window 3, which corresponds to lipoproteins and phosphate compounds, ranges from 1500 to 1200 cm^–1^. In *N. brasiliensis*, bending vibrations of -CH_2_, symmetric vibrations of COO groups at 1402 cm^–1^, amide III and asymmetric vibrations at 1250 cm^–1^ of *P* = O groups were detected. Window 4 ranges from 1200 to 900 cm^–1^ and corresponds to polysaccharides; *N. brasiliensis* signals were detected at 1080 cm^–1^, corresponding to C-O-C, and at 950 cm^–1^, corresponding to C-OH. Finally, window 5 shows the fingerprint region from 900 to 400 cm^–1^; here, *N. brasiliensis* signals of C-OH were detected at both 850 and 530 cm^–1^. The spectrum also showed broad signals at 3270, characteristic of O-H for the biomolecules present in this bacterium. It is worth mentioning that there were no previous reports on the FT-IR spectrum of *N. brasiliensis*.

## 4. Discussion

### 4.1. Metabolism of *Nocardia brasiliensis*

**FIGURE 12:**
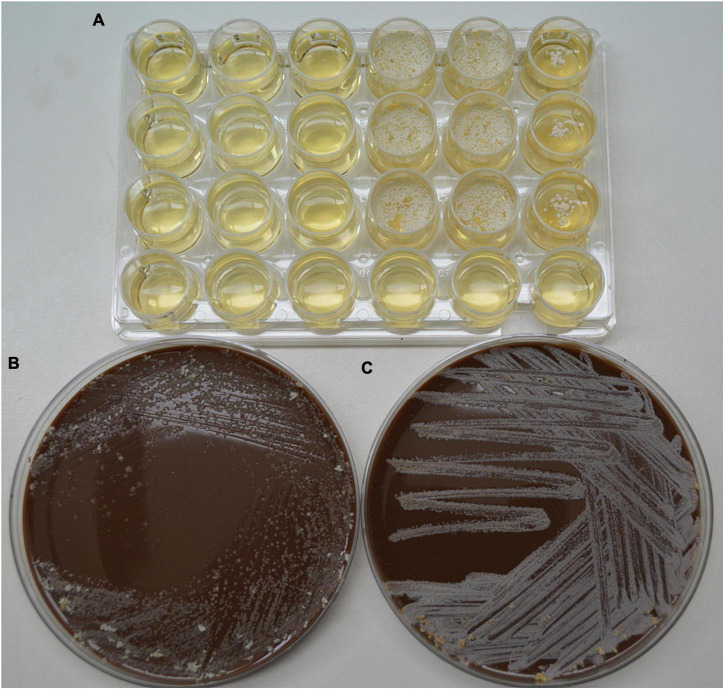
Comparison of the growth of control strain HUJEG-1 and FM-825 under the effect of β-HFC. **(A)** 24-well plate with Mueller-Hinton + β-HPC broth; 5 × 10^5^ CFU/mL *N. brasiliensis* HUJEG-1 (ATCC 700358) was seeded in nine wells from the first three columns: 1–3, and rows **(A–C)**. In column 3 no β-HPC was added, and after 7 days of incubation there was no growth. A total of 5 × 10^5^ CFU/mL of *N. brasiliensis* FM-825 was seeded in columns 4–6 and rows **(A–C)**. Column 6 was not added with β-HPC. Growth was observed in the seeded wells, and those without β-HPC had little growth. Row D was used as a sterility control of the procedure. **(B)** Chocolate agar plate, 5 × 10^5^ CFU/ml of *N. brasiliensis* HUJEG-1 (ATCC 700358) were seeded after thawing, grown in M-H with β-HPC and standardized with the McFarland Nephelometer; in all areas of striation, little growth is observed. **(C)** Chocolate agar plate, 5 × 10^5^ CFU/mL of *N. brasiliensis* FM-825 were seeded after thawing, grown on M-H agar with β-HPC and standardized with the McFarland Nephelometer; abundant growth is observed in all areas of striation.

As previously stated, the hydrolysis of *N. brasiliensis* can be seen with the naked eye. At the clinical level, the hydrolase activity, morphological characteristics, and growth lapse are good enough to identify it at the species level. Catabolism is dictated by enzymes that are genetically encoded (Trevino-Villarreal et al., 2012). Some are localized in ERS1247828SC, the NCTC11294 strain. As an oxidizer microorganism, *N. brasiliensis* obtains energy primarily through the tricarboxylic acid cycle (TCA), which releases energy from acetyl CoA. In our experimentation, glucose was not directly provided; instead, proteins and lipids were metabolized by Nocardia. Lipases break down lipids as carbon sources to harvest acetyl CoA and glycerol. Through the hydrolysis of peptide bonds, proteins are broken down into amino acids, and each newly formed intermediary will participate in glycolysis to include pyruvate or acetyl CoA. In eukaryotic cells, L-carnitine is a substance essential for transporting long-chain fatty acids through the inner mitochondrial membrane: the “carnitine shuttle” to β oxidation. So far, *N. brasiliensis* can metabolize fatty acids and carnitine in a sequential metabolic pathway. In bacteria, β-oxidation is an alternate way to obtain pyruvate, as previously reported in *Escherichia coli* (Adamus-Białek et al., 2017). *In silico* analyzes have shown that *N. brasiliensis* has an acyl-CoA dehydrogenase fadE12 NCTC11294_01240, which probably binds FAD as an oxidoreductase. In addition, it has a putative acyl-CoA dehydrogenase (AFU05542) that could bind flavin; as part of the oxidation-reduction process. The alpha subunit encoded in 3 genes: 3-hydroxybutyryl-CoA dehydrogenase (fadB2_3NCTC11294_07044), would participate in the next step of fatty acid oxidation by NAD + reduction. A family of 10 subunits could function as an enoyl-CoA hydratase (echA8_1 NCTC1129294_00702), another enoyl-CoA hydratase (O3I_029395), and an enoyl-CoA hydratase/isomerase that adds water to prepare the substrate for the next step by reducing NAD^+^ and thus favoring electron transport for energy production. The last step of β-oxidation would be performed by a fadA complex of three Rv0859 acyltransferases (fadA_2 NCTC11294_01893, fadA_3NCTC11294_02176, and fadA_5 NCTC11294_03883). According to the β-oxidation pathway (fadA_6 NCTC112929294_05772), which is a 3-hydroxy acyl-CoA dehydrogenase (O3I_001385) prepares the substrate by reducing NAD^+^. Finally, we assume that the enzyme 3-ketoacyl-CoA thiolase (fadA_1 NCTC1129294_01403) present in the bacterium will generate acetyl CoA (for TCA) and acyl CoA to return to the first step of β-oxidation (DiRusso, 1990). Furthermore, *N. brasiliensis* also has a protein (fadJ NCTC11294_01894) involved in the β-oxidation of anaerobic microorganisms (Rogers et al., 2014), which is needed to investigate its participation in the metabolism of *N. brasiliensis* since the bacterium is strictly aerobic. L-carnitine can be supplemented as a unique carbon source, and bacteria transform it into pyruvate. In our experiment, the β-hydroxy-phosphonate boosted metabolic activity. *In silico*, *N. brasiliensis* possesses the counterpart enzymes related to the catabolism of L-carnitine that have already been described in *E. coli*. The intake may be mediated by an ATP-binding cassette transporter (F5 × 71_07380). Acylcarnitine hydrolase is not reported to obtain γ-butyrobetaine, nor is γ-butyrobetaine hydroxylase noted; both are needed to split carnitine. In *N. brasiliensis* enzymes related to *E. coli* anaerobic carnitine catabolism were found in two ways: L-carnitinyl-CoA dehydratase (Carnitinyl-CoA dehydratase) (caiD_1-10 NCTC11294) and a crotonase/enoyl-CoA hydratase family protein (WP_172595718) that in *E. coli also* has the function of carnitine hydratase and racemase (gene caiD) (Bullock et al., 2018). There was also a carnitine operon protein CaiE (yrdA_1 NCTC11294_03304), whose function has yet to be determined since the enhancement of catabolic activity achievement was obtained. In fact, among the strains of *N. brasiliensis*, some differences must be addressed. Depending on the previous environment of residence or treatment relative to the nature of the substrate. *N. brasiliensis* FM-825 has been used to infect and reinfect mice (Millán-Chiu et al., 2011); Once obtained from infected mice, it was subjected to hydrolysis experiments, while *N. brasiliensis* HUJEG-1 (ATCC 700358) had been frozen, thawed, and seeded cyclically in BHI broth and agar as a control of the biochemical metabolism of the experimental strain. Both strains were seeded (5 × 10^5^ approximately CFU/ml) in a 24-well plate of MH broth with and without β-HPC; also were seeded chocolate agar plates seeded were also considered as nutrient medium. There was sustained growth of FM-825 with β-HPC at the end of the seventh day of incubation. The strain HUJEG-1 (ATCC 700358) did not grow in that time frame, demonstrating that the bacteria need more time for adaptation and enriched media to grow after thawing to operate metabolic pathways in response to the L-carnitine analog (Figure 12). Despite the above, the metabolically active strain is an example that, with the help of β-HPC, could serve as a biotransformation enhancer, as it can cleave the aromatic ring of L-tyrosine. The Nocardia genus has attracted the attention of biotechnologists for its ability to produce metabolites with pharmacological uses (Barrow and Feltham, 1993; Genilloud, 2017; Wink et al., 2017; Dhakal et al., 2019). For example, *Nocardia uniformis* subsp. *tsuyamanensis* produces nocardicins with antimicrobial activity, and *Nocardia pseudobrasiliensis* produces nocardiothiocines with antimicrobial and antineoplastic activity. *Nocardia farcinica* produces nocobactins and siderophores that facilitate the uptake of metal ions, such as iron, and can be used as modulators of metabolic pathways to block or allow growth (Aguado-Santacruz et al., 2012). Similarly, several species of Nocardia are known to produce multiple enzymes and metabolites that can be exported to the surrounding environment.

### 4.2. Casein and l-tyrosine catabolism

Casein is a protein complex formed by α-casein joined to β-casein or κ-casein that, once bounded, conform micelles that prevent calcium precipitates in the biological form suspended as milk. Forces such as hydrophobic and electrostatic bonds are also present; hydrogen bond bacteria cause the rupture and, once initiated by the rupture, the looseness of interactions make the different casein molecules available to be hydrolyzed using the carbon molecules and lactose as energy source (Bhat et al., 2016). As shown in Figure 2, differences in hydrolysis were observed, the addition of β-HPC to the substrate allowed bacteria to improve their metabolic capacity through the accumulation of hydroxyl groups (OH), presumably by rupture of the peptide bonds. In addition, carbonyl groups (*C* = O) and phosphates were added as signatures of increased hydrolysis; the least squares and statistical summaries made evident that hydrolysis significantly increased. The goal was to quantify the remains obtained by comparing them with the same substrate subjected to five double dilutions from 20 to 1.25 g in a calibration curve where we could place the hydrolyzate of *N. brasiliensis* and *N. brasiliensis* + β-HPC at a closeness of 1.25 g casein. Both experimental conditions appeared similar (Figure 6A); therefore, it was necessary to perform a statistical analysis to find the difference between them. The principal component analysis (PCA) found differences among the experimental substrates; the hydrolyzate of *N brasiliensis* (0.393163) approached 20 g casein, and *N. brasiliensis* + β-HPC was out of the calibration curve since the eigenvalue was the lowest at 0.3008444. These results allowed us to propose the β-HPC as a metabolic enhancer or modulator of bacterial activity. For example, it is desirable to control the activity of thermoresistant proteases of *Pseudomonas aeruginosa* over casein in order to obtain better cheese (Paludetti et al., 2020) and to conjugate an extensively hydrolyzed casein formula that includes *Lactobacillus rhamnosus* to prevent cow’s milk allergy in infants (Berni Canani et al., 2017). Plants such as *Dregea sinensis* can provide proteases capable of extracting peptides from milk with antimicrobial activity; these peptides are meant to prevent dairy products and food from rotting (Zhao et al., 2020). We have directed our study toward a different use of *N. brasiliensis* metabolism, not only as an accidental pathogen, but also as a biotechnological tool that can be applied to the dairy industry.

We know that *N. brasiliensis* hydrolyzes **L-tyrosine** (Scheuer, 2014; Fatahi-Bafghi et al., 2016), as confirmed by FT-IR in Figure 3, where the orange line indicated heavier but smooth peaks owing to the accumulation of free OH previously attached to the aromatic ring as a radical group (Fuchs, 2008), which was seen by comparing the L-tyrosine substrate spectrum represented in blue. This activity was compared with *N. brasiliensis* + β-HPC, and the highest transmittance is shown in red (Figure 2). and confirmed in the calibration curve, L-tyrosine is a strongly fluorescent amino acid, and a simple cleavage of bond tyrosyl amide bond produces an approximately twofold increase in fluorescence monitored with spectrophotometry. It is precisely the aromatic ring that acts as a resonance energy acceptor (Peranteau et al., 1995). The increases in transmittance were reflected and quantified in the statistics, especially in APC, *N. brasiliensis* (0.3663); *N. brasiliensis* + β-HPC (0.3735). The amino acid L-phenylalanine is valuable as the precursor of melanin and thyroxine. It can be broken down through tyrosine decarboxylase (TDC) to obtain tyramine, a monoacid that holds the phenolic ring and is a frequent flavoring additive in food; nonetheless, it can cause food poisoning and contribute to the wastewater caused by amino acid synthesis (Marcobal et al., 2012; Fatahi-Bafghi et al., 2016; Zhang et al., 2016). L-tyrosine can also be broken down to fumarate and acetoacetate with a tyrosine transaminase, and *N. brasiliensis* has a serine hydroxymethyltransferase (glyA1_1 glyA, NCTC11294_00384), a tyrosine recombinase XerC and XerD (xerC_2 xerC, NCTC11294_06080, xerD NCTC11294_03835), tyrosine protein phosphatase (iphP_3 NCTC11294_06441). Another enzyme with transaminase activity was found in the genome of *N. brasiliensis*, which confirmed the regular catalysis of this amino acid. To split the aromatic ring, a homogentisic acid 1.2-dioxygenase is necessary; *N. brasiliensis* harbors an acid reductase dioxygenase (mtnD NCTC11294_00067) and four non-specific dioxygenases (NCTC11294_03881, NCTC11294_03172, NCTC11294_05468, NCTC11294_01111). The aromatic ring split occurred at the signals at 3050 cm^–1^ and between 800 and 700 cm^–1^ disappeared; these signals corresponded to double bonds and aromatic rings. This was achieved by *N. brasiliensis* + β-HPC. It is important to identify *N. brasiliensis* enzymes that are enhanced by β-HPC so that it can be used broadly in pharmacological activities; for example, the benzene ring of L-tyrosine can be hydroxylated and prenylated to generate l-3,4-dihydroxyphenylalanine (L-DOPA), which is part of the treatment of Parkinson’s disease (Tan et al., 2020).

### 4.3. Tween 80 and egg yolk catabolism

**Tween 80** is a synthetic polyethylene sorbitol ester. Oleic acid is the primary fatty acid; the compound is water-soluble and applied as a surfactant, solubilizer, and additive in the food and pharmaceutical industries, as well as in medicine for the isolation of mycobacteria (Larson et al., 2020; Tween 80, 2022). *N. brasiliensis* is seeded in Tween 80 broth to induce the bacteria to grow as a homogeneous suspension instead of the tight layer that usually extends on the surface of the brain heart infusion. The structure can be hydrolyzed when the ester is unbounded to the sorbitan head. In addition, oxidation of double bonds can occur with the formation of peroxides, aldehydes, and ketones (Larson et al., 2020). *N. brasiliensis* has a set of catalase-peroxidases (katG NCTC11294_03982) and oxidases that possibly have activity in alkenes to obtain aldehydes. These compounds can also be obtained through a predicted esterase (NCTC11294_00337) that reduces carboxylic acid to form aldehyde. It is notable for the strong signal at 2900–2800 cm-1 in *N. brasiliensis*; such signals disappeared in *N*. *brasiliensis* + β-HPC. Their activity was probably enhanced by these enzymes or perhaps another kind of enzyme such as lipase 3 (lip3 NCTC11294_07104), and secretory lipase (NCTC11294_03890) entered to break the esters and finally utilized the structural carbons, leaving a faint signal as proof of substrate exhaustion. In 1100 cm^–1^, a strong signal from esters belonging to *N. brasiliensis* contrasted with a lack of signal from *N. brasiliensis* + β-HPC that consumed the substrate; in this case, the L-carnitine analog improved the metabolism of lipids, using them as a carbon source (Figure 3). Such differences can be observed in the transmittance from the calibration curve. *N. brasiliensis* increased this hydrolysis to 2%; however, greater hydrolysis from *N. brasiliensis* + β-HPC was obtained as indicated in PCA: the control substrate had an eigenvalue of 0.3946757 corresponding to the concentration of 0.0125%; *N. brasiliensis* had an eigenvalue of 0.3959531, which means proximity to the chosen control. The *N. brasiliensis* + β-HPC eigenvalue was 0.2651606; this value ended below without finding a match inside the curve (Figure 6C and Table 2). Bacteria use lipases to break down lipids in their environment, whether belonging to the host or are supplemented in culture media for the acquisition (Chandra et al., 2020). Apparently, in the host, this activity improved the inflammatory response. *Pseudomonas aeruginosa* accelerated lung destruction by hydrolyzing esterified fatty acids within the pulmonary surfactant, *Propionibacterium acnes* secreted lipases that cleaved sebum triacylglycerides into glycerol and free fatty acids that ultimately caused inflammation in the sebaceous follicle (Chen and Alonzo, 2019). We ask ourselves: If a host is infected with *N. brasiliensis* fueled by β-HPC, would the mycetoma become more destructive or the infection become systemic? This question must be addressed (Zijlstra et al., 2016). Yet again, as Chandra, Singh, and Arora quote: “Microbial lipases are universal in nature and are commercially substantial due to their low manufacturing cost, superior stability, and more availability than animal and plant lipases”. Natural or recombinant microbial lipases are generally used in diverse bioengineering applications (Chandra et al., 2020).

If there could be an “all-in-one food,” it would be the egg. In the **yolk**, lipids and fat can reach 32.5%, proteins 17.5%, minerals 2%, and the remaining content is water (Kusum et al., 2018). In our experiment, the behavior was similar to that of the casein substrate because, instead of disappearing, the signal was strong and pronounced (Figure 5). Two phases comprise the yolk: granules and plasma; The granules are formed by high-density lipoproteins (HDLs) assembled into soluble micelles of 100–200 nm, highly resembling casein granules. There are also low-density lipoproteins (LDL) composed of nanoparticles (17–60 nm), phosvitin, and other proteins (Ovalles, 2015; Kusum et al., 2018). It was not possible to determine which compounds were hydrolyzed. A signal of OH at 3300 cm^–1^ was detected; these compounds are mainly part of proteins but are yet been verified. *N. brasiliensis* has extensively proven proteolytic activity and at 2900 cm^–1^, two peaks: CH_3_ and CH_2_ were at the substrate level in *N. brasiliensis* and were fueled by β-HPC. Since growth and hydrolysis occurred, these compounds may be aldehydes and ketones, mostly from proteins that make up 50% of yolk plasma; some are attached to carbohydrates whose content is 0.5%. Most of the yolk carotenes are in plasma, almost 1.0%. They are lutein, zeaxanthin, and β-carotene. They can be free as part of fatty acid esters that make them easily accessible. Therefore, the stronger signal of β-HPC equalized the hydrolysis of such compounds at 1650 to 1400 cm^–1^, but perhaps the phosvitin catabolism that has a remarkable resistance to hydrolysis, thanks to the β-HPC the amide II stretching vibration increased, as well as the heavier phosphate signals at 1071 and 520 cm^–1^, also a signal of increased hydrolysis (Huang et al., 2019; Zhao et al., 2021; Figure 5, 6D). *N. brasiliensis* + β-HPC consumed proteins, lipids and carotenes, as witnessed by the colorless supernatant after growth, compared to *N. brasiliensis*, which maintained a yellowish hydrolyzate (data not shown) (Martínez-Robles et al., 2019). In this particular substrate, statistical summaries placed *N. brasiliensis* at 187 μL and *N. brasiliensis* + β-HPC at 93.75 μL instead of PCA. In the biotechnology field, much has to be done; hydrolyzates can also chelate calcium and retain antibacterial activity, as has been seen in *E. coli* (Yilmaz and Ağagündüz, 2020). Despite availability, egg consumption has declined due to the high cholesterol and fat content. If targeted hydrolysis can be achieved, purified peptides can make the egg a major option for nutritional food (Chang et al., 2018).

### 4.4. FT-IR characterization of *N. brasiliensis*

When we performed FT-IR catabolism studies of *N. brasiliensis* with and without β-HPC with the substrates mentioned above, we searched for the spectrum of this bacterium to compare the behavior. We found a *N. brasiliensis* spectrum (Asai et al., 1963) it was useful since there were strong coincidences aiding us to characterize *N. brasiliensis* by FT-IR. In (Figure 11) in the window of lipids of the interval 2800–3000 cm^–1,^ signals are found at 2870, 2930, and 2970 cm^–1^ that correspond to alkanes. In the protein window 1500–1800 cm^–1^, we find the amide I and amide II signals mainly at 1550 cm^–1^ (Jinghang et al., 2022). In the nucleic acid window 1200–1500 cm^–1^, we found a COO signal at 1400 cm^–1^, and in this same window, a signal at 1250 cm^–1^ corresponds to phosphates. In the carbohydrate window 700–1200 cm^–1^, we found ether signals. Finally, we report an intense C-OH signal in the area of the fingerprint, which is not analyzed in the above-mentioned work (Asai et al., 1963). Were detected with greater intensity and the fingerprint signal was more defined than reported for other bacteria (Helm et al., 1991; Naumann et al., 1991; Novais et al., 2019; Rodrigues et al., 2020; Passaris et al., 2022).

## 5. Concluding remarks

β-HPC is suitable for use as an additive in bacteria culture media, since it enhances metabolism; it increases the retrieval of CFUs and bacterial hydrolytic activities.

*Nocardia brasiliensis* can no longer be considered exclusively as an opportunistic intracellular pathogen. The bacterium is capable of producing metabolites that must be analyzed, and β-HPC plus the bacterium can also be used as biotechnological alternatives in pharmacology, bioremediation, and dairy elimination subproducts.

FT-IR technology has already been widely used as a bacterial typifying and diagnostic aid. *N. brasiliensis* can be recovered from patients through grains that can be washed, crushed, and dried on glass slides to expose them to FT-IR or seeded in culture media as soon as they grow as a compact colony. It is easy to manipulate and immobilize on a surface to obtain FT-IR spectra, accelerating diagnostics and avoiding time-consuming identification tests. Here, we offer the spectrum of *N. brasiliensis* FM-825 isolated from a mycetoma patient as a comparative aid; several *N. brasiliensis* strains must be processed with FT-IR to establish them as diagnostic tool.

## Data availability statement

The raw data supporting the conclusions of this article will be made available by the authors, without undue reservation.

## Author contributions

SM-R and EG-B: conceptualization. SM-R: methodology, investigation, and writing – original draft preparation. AT-J: software. GV and BJ-L: validation and writing – review and editing. GV: formal analysis. EA and JR-E: resources. IT-T and EG-B: visualization. VV-V and EA: supervision. JR-E: have an insightful mind full of keen ideas that nourished this work, passing left us with deep sorrow; however, his teachings, expertise, and sweetheart make us honor his loving memory. All authors contributed to the article and approved the submitted version.
